# First identification of a patent pentastomid pulmonary (*Raillietiella orientalis*) infection in a captive Meller's chameleon (*Trioceros melleri*) in Germany

**DOI:** 10.1016/j.ijppaw.2025.101045

**Published:** 2025-02-03

**Authors:** Paula Sapion-Miranda, David Ebmer, Edwin Kniha, Julia Walochnik, Saskia Dreyer, Dominik Fischer, Lisa Grund, Anja Taubert, Carlos Hermosilla, Malek J. Hallinger

**Affiliations:** a Institute of Parasitology, Justus Liebig University Giessen, Giessen, Germany; b Exomed GmbH, Marburg, Germany; c Vienna Zoo, Vienna, Austria; d Institute of Specific Prophylaxis and Tropical Medicine, Center for Pathophysiology, Infectiology and Immunology, Medical University of Vienna, Vienna, Austria; e Der Grüne Zoo Wuppertal, Wuppertal, Germany; f Zoom Erlebniswelt Gelsenkirchen, Gelsenkirchen, Germany

**Keywords:** Chameleons, Pentastomids, Reptile medicine, Wildlife, Parasite spillover, Emerging diseases

## Abstract

A female, zoo-housed, adult Meller's giant one-horned chameleon (*Trioceros melleri*) showed clinical symptoms including halitosis, obstipation, dysecdysis and shed pentastomid eggs with the faeces. After a patent pentastomiasis was diagnosed, the chameleon's condition worsened after repeated ivermectin treatments, and the animal was euthanized due to animal welfare reasons. The necropsy revealed that the lungs were infected with 29 adult pentastomid specimens. Based on morphological and ultrastructural characteristics pentastomids were identified as *Raillietiella orientalis*. Additionally, this species identification was confirmed by DNA sequencing (18S rRNA).

Pentastomid-infected insects, such as cockroaches, might play an important role in the transmission of *R*. *orientalis* as suitable obligate intermediate hosts. Another crucial factor to be considered is the importation of wild caught individuals, providing a potential source for numerous emerging infectious disease transmissions and parasite spillovers.

This is the first report, to the best of our knowledge, on a patent pulmonary *R. orientalis* infection in a captive chameleon. We call for further investigations on neglected pentastomid infections in chameleons and lizards kept as pets to better understand implications of this new host record and its possible role in transmission of emerging pentastomiasis.

## Highlights

• Invasive pentastomid *Raillietiella orientalis* in a Meller's chameleon (*Trioceros melleri*).
• Patent pentastomiasis confirmed by egg shedding/identification of adult pulmonic pentastomids.
• Limited drug therapy-efficacy of pentastomid infection leading to euthanasia of the chameleon.

## Graphical abstract

**Figure:**
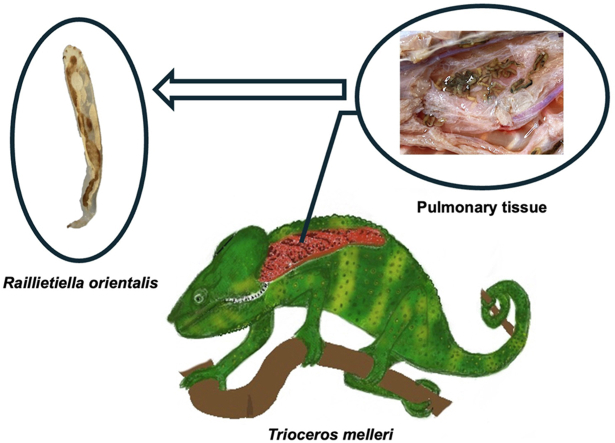

## 1 Introduction

Reptiles in captivity are susceptible to various endoparasitic infections, including pentastomiasis (Deakins, 1972; Paré, 2008; Hallinger et al., 2020).

Pentastomids are endoparasitic arthropods residing as adults in the respiratory tracts, primarily in the lungs of vertebrates, acting as definitive hosts (DH), with most of them being squamate reptiles (Riley, 1986; Lavrov et al., 2004). Most pentastomids exhibit an indirect (heteroxenous) lifecycle, relying on various intermediate hosts (IH) which can be rodents, lizards, anurans, fish and insects (Lavoipierre and Rajamanickam, 1973; Ali and Riley, 1983; Haugerud, 1989; Kelehear et al., 2014; Barton and Morgan, 2016). Members of the order Porocephalida also make use of mammals, such as cattle, rabbits or marsupials, as IH (Riley et al., 1985; Paré, 2008; Barton et al., 2020b, 2020c, 2022).These IH become infected after ingestion of embryonated eggs and within IH the larvae hatch and undergo multiple molts to become an infective nymph (Riley, 1986; Boyce and Kazacos, 1991; Paré, 2008; Tappe, 2009). Upon ingestion of nymph-carrying IH by the DH, these nymphs actively migrate to the lungs thereby completing their life cycles (Ali and Riley, 1983; Riley, 1986; Paré, 2008). In some pentastomid species, a direct lifecycle is suspected (Self, 1969; Riley, 1972; Spratt, 2003; Paré, 2008).

*Raillietiella* is a genus within the pentastomid order Cephalobaenia, infecting squamate hosts and frequently found in snakes and wild-caught geckos but also skinks, varanids, and agamas (Paré, 2008;Wellehan and Walden, 2018). The species *Railletiellla orientalis* (Hett, 1915) is endemically widespread in Asia in a variety of snakes of the families Colubridae, Elapidae, Viperidae and Boidae (Ali et al., 1985; Christoffersen and De Assis, 2013; Walden et al., 2020). Recently, *R*. *orientalis* has newly been found as neozoan parasite spreading across North America and establishing in the US State of Florida in at least 14 native snake species (Farrell et al., 2019; Miller et al., 2018; Walden et al., 2020). Furthermore, *R. orientalis* infections have been described in Argentinian black and white tegus (*Salvator merianae*) (Fonseca, 2021; Goetz et al., 2021) as well as in Tokay geckos (*Gekko gecko*) (Fieldsend et al., 2021) and occurring in native snakes of Australia (Kelehear et al., 2014; Barton and Trembath, 2024).

This report documents a clinical case of infection caused by *Raillietiella orientalis*, confirmed through both morphological and molecular analysis. These findings aim to raise awareness of an under-investigated parasite.

## 2 Material and methods

### 2.1 Case details, treatment and course of condition

An adult female Meller's giant one-horned chameleon (*Trioceros melleri*) was kept for 4.5 years (since 2016) at a zoological garden in Germany, with a history of being captured from the wild in Tanzania years ago and being kept as pet between capture and donation to the zoo. The animal was fed live migratory locusts (*Locusta migratoria*) and crickets (*Gryllus bimaculatus*), with additional supplementation of vitamins, minerals, and amino acids (Korvimin ZVT®, WDT, Garbsen, Germany). It inhabited a tropical enclosure measuring 4.60 x 2.00 x 3.00 meters (length x width x height). For over a year, it was kept together with a male conspecific for breeding purposes but had no breeding success. The male animal was later returned to its owner and died in 2021, with no evidence of pentastomid infection. Notably, the zoo experienced a persistent hygiene issue with cockroaches (*Blatella germanica*) within this building, and despite constant pest control measures, cockroaches were frequently observed within the chameleon's enclosure. The medical history of the animal includes a right mandibular fracture associated with halitosis and dysbacteria, moreover an issue of obstipation and dysecdysis. Routine parasitological examinations of the faeces were performed at least bi-annually and revealed a *Trichomonas* spp. infection in 2017 and 2018, which was subsequently treated with a series of 35 mg/kg metronidazole administered orally over 5 days. Previously, in 2018, pentastomids have been considered by the zoo veterinarians, but only tapeworm eggs were identified in an external laboratory, and the animal was treated with praziquantel. In 2019, a faecal sample was diagnosed as positive for *Heterakis* spp. eggs by a commercial laboratory and thereafter subcutaneous levamisole (0,5 mg/kg body weight; Quadrosol 10%, animedica Herstellungs GmbH, Senden-Bösensell, Germany) followed by an oral fenbendazole suspension (50 mg/kg body weight; Panacur 10%, MDS Sharp & Dohme GmbH, Munich, Germany) the treatment was initiated. In January 2021 pentastomid eggs were discovered by exomed GmbH in a routine faecal examination. When the diagnosis of pentastomiasis was finally confirmed, hygiene and personal protection measures were enhanced beyond the mandatory basic hygiene practices, such as washing hands after working with animals. Additional precautions included wearing disposable gloves when handling the animal or its enclosure equipment, since a potential zoonotic risk was unclear. Furthermore, the care routine was adjusted so that the terrarium of the affected animal was attended to last by the responsible keepers, to reduce the risk of transmission to other enclosures.

Following the confirmation of pentastomiasis, the chameleon received subcutaneous ivermectin as treatment (0.2 mg/kg; Ivomec®, 1% ad Inj., Boehringer Ingelheim Vetmedica GmbH, Ingelheim am Rhein, Germany) alongside a supportive care regimen, fluids, anti-inflammatory drugs, and vitamins. Six days after the initial treatment, the chameleon showed acute dyspnea and severe apathy but gradually improved after receiving oxygen, antibiosis and supportive care. Eventually, a second ivermectin therapy was administered 14 days after the initial treatment, and this time, the chameleon did not display any worsening symptoms. On the contrary, the animal gained weight and improved general body condition. However, a control faecal exam conducted six weeks after the treatment still showed a patent pentastomid infection with high-grade egg shedding.

Unfortunately, the chameleon's condition deteriorated after a third ivermectin treatment at the end of March 2021. Despite continued supportive care, the chameleon failed to recover and was euthanized with pentobarbital IV (400 mg/kg, Release® 500mg/ml, WDT, Garbsen, Germany) one week after the third ivermectin injection.

### 2.2 Pathological examination

A necropsy was conducted one day later at exomed GmbH, Marburg, Germany, to investigate the underlying health issues according to Farris et al. (2015).

### 2.3 Pentastome identification, DNA extraction, amplification, and scanning electron (SEM) analysis

Adult pentastomids were extracted from the lung tissue and preserved in 70% ethanol to further on being examined morphologically and molecularly for species identification. Pentastomes were primarily identified based on morphological characteristics of male spicules, which feature a prominent spicula base marked by a raised reticulum, posterior hooks that are larger than the anterior pair and the triangular-shaped cephalothorax, as described in previous reports (Ali et al., 1985; Riley, 1986; Kelehear et al., 2011; Walden et al., 2020).

DNA was isolated from an individual specimen with a QIAamp® DNA Mini Kit 250 (Qiagen, Hilden, Germany) with a final elution volume of 200 μL (μL). For molecular characterization, a fragment of the small subunit ribosomal ribonucleic acid (ssU rRNA) gene was amplified by PCR using the primer combination Pent629F/Pent1011R with an expected length of 380–400 basepairs (without primers) depending on the pentastomid species, based on the published protocol by Brookins et al. (2009). Amplification by PCR was performed with an Eppendorf Mastercycler (Eppendorf AG, Hamburg, Germany) in 10 x reaction buffer B with 2.5 mM MgCl2, 1.6 mM nucleoside triphosphate (dNTPs), 1 μM primers, 1.25 units DNA polymerase (Solis BioDyne, Tartu, Estonia) and 5 μL DNA. Sterile H_2_O was added to a final volume of 50 μL. PCR conditions were adapted to the following protocol: 95 °C for 15 min, followed by 40 cycles of 95 °C for 30 s, 58 °C for 1 min and 72 °C for 1 min, followed by a final extension at 72 °C for 10 min.

Bands were analyzed with a Gel DocTM XR + Imager (Bio-Rad Laboratories, Inc., California, U.S.A.), cut from the gel and purified with an IllustraTM GFX™ PCR DNA and Gel Purification Kit (GE Healthcare, Buck-inghamshire, UK). Sanger sequencing was performed with a Thermo Fisher Scientific SeqStudio (Thermo Fisher Scientific, Massachusetts, USA). Sequences were obtained from both strands and a consensus sequence with a length of 382 basepairs was generated in GenDoc 2.7.0 and uploaded to GenBank (Accession number: PQ518867).

For SEM analysis**,** one adult female specimen was selected for acquisition of ultrastructural images. The specimen was fixed in 2.5% glutaraldehyde and post-fixed with 1% osmium tetroxide (both Merck, Darmstadt, Germany), rinsed in distilled water, dried, CO_2_-treated to the critical point, and sputtered with gold particles. SEM analysis was conducted with a Philips XL30® scanning electron microscope equipped with a digital camera.

## 3 Results and discussion

**Fig. 1:**
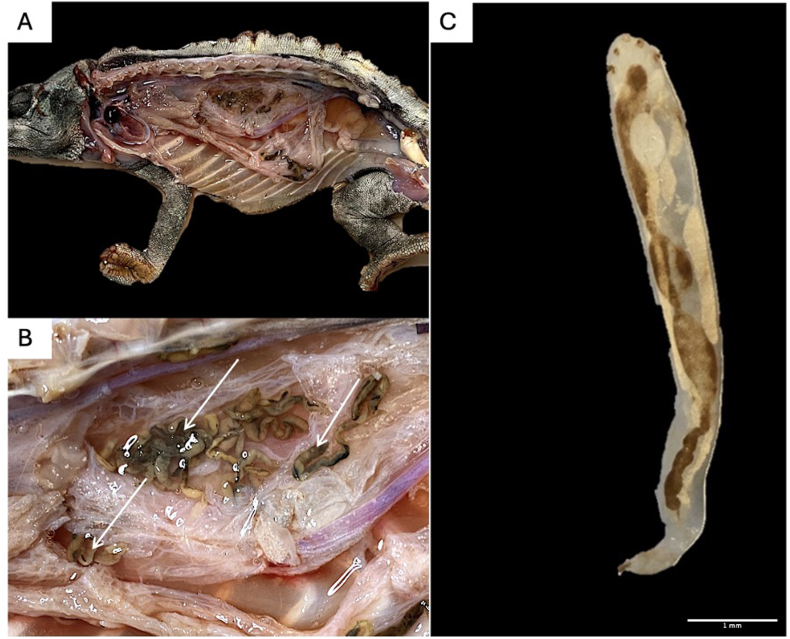
A. Lateral view of dissected Meller's chameleon (*Trioceros melleri*): Severe infection with adult pentastomids in the lung tissue **B.** Lung tissue close up with adult pentastomids (indicated by white arrows) **C.** Single adult pentastomid *Raillietiella orientalis*.

**Fig. 2:**
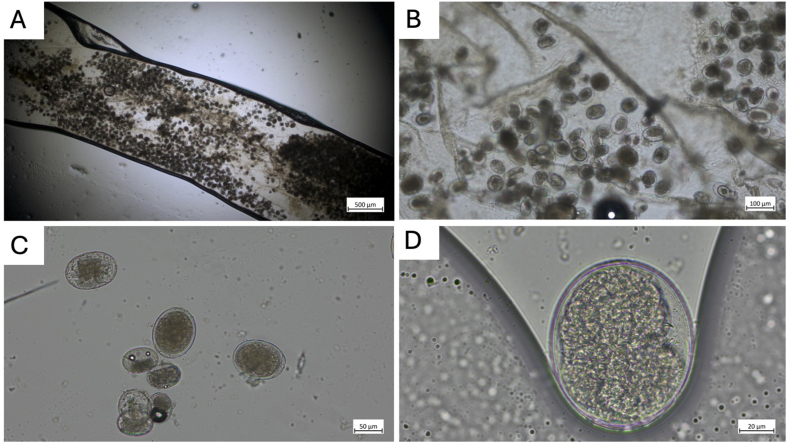
A. Gravid female *Raillietiella orientalis***B.** Close-up view of uterus containing eggs **C.** Eggs extracted from the uterus **D.** Single egg recovered from gravid *R. orientalis*.

The necropsy confirmed the pentastomid infection with a total of 29 adult lifeless pentastomid specimens (*n* = 29) recovered from the lung tissue of the chameleon (Fig. 1). A *post-mortem* faecal examination revealed a substantial presence of pentastomid eggs (Fig. 2). In the lung tissue, the faveoli contained detritus and numerous pentastomid eggs, with the tissue appearing almost bloodless. Intercoelomic organs exhibited multifocal euthanasia artefacts, necrotised enteritis on the entire length of the intestine was observed. The liver showed histopathologically vacuolated degeneration of hepatocytes, diffuse siderophages, multifocal microgranulomas and single foci of necrosis, possibly due to pentastomid migratory nymphs. Additionally, bacterial isolation from the liver disclosed a heavy growth of *Pseudomonas synxantha, Enterococcus faecalis,* and a moderate growth of *Enterococcus casseliflavus,* suggesting a secondary infection.

**Fig. 3:**
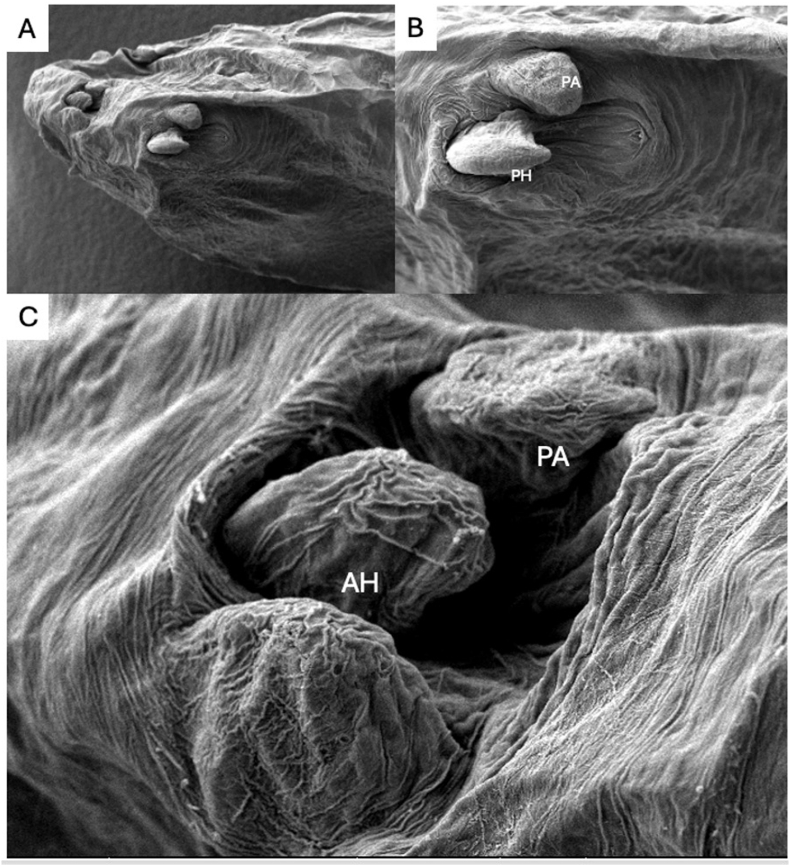
Ultrastructural scanning electron microscopy (SEM) images of *Raillietiella orientalis* anterior part collected from a Meller's chameleon (*Trioceros melleri*). **A.** Cephalothorax of the pentastome, ventral-lateral view. **B.** Closer ventral view unveiling the sharp posterior hook (PH) with cephalic papillae (PA) and hook pit **C.** Lateral view of anterior hook (AH) with prominent cephalic papillae (PA) beside.

Comparison to available sequences in the GenBank database using the Basic Local Alignment Search Tool (BLAST) revealed 100% identity with *Raillietiella orientalis* extracted from e.g. *Pantherophis guttatus* (MG559593), *Sistrurus miliarius* (MK072952), and *Gekko gecko* (MW692107). For a detailed depiction of the hooks and overall pentastome morphology, SEM images were taken (Fig. 3).

To the best of our knowledge, this is the first report of *R*. *orientalis* in a Meller's chameleon (*T. melleri*) and across the chameleon taxa. Over the last decades through increased illegal or legal international exotic pet trade, pentastomids have been also introduced through various continents such as Australia (Barton, 2009; Kelehear et al., 2014; Barton and Trembath, 2024), North America (Farrell et al., 2019), Central America (Kelehear et al., 2015) and Europe (Mendoza-Roldan et al., 2022). In the USA, this neozoan pentastomid species, is nowadays rapidly spreading in the US State of Florida, posing a serious threat to the native snake population (Miller et al., 2018; Walden et al., 2020).

Initially, *R*. *orientalis* was predominantly associated with snakes, while small lizards believed to act primarily as IH (Ali et al., 1985; Kelehear et al., 2014; Walden et al., 2020). However, this observation of adult pentastomids in the chameleon's lung, along with the detection of ova shedding, have reaffirmed the observation of Bosch (1986), who described frogs and cockroaches as more suitable IH of this parasite (Bosch, 1986). Meanwhile, lizards such as geckos (Fieldsend et al., 2021), tegus (Fonseca, 2021; Goetz et al., 2021) and in this presented case - chameleons - are suitable as DH for this parasite.

Insects, such as the presence of cockroaches in zoos, have been reported to be able to significantly contribute to the transmission of pentastomids (Wright, 1997; Bosch, 1986; Mendoza-Roldan et al., 2022) as observed with other parasites such as *Hexametra angusticaecoides* (Barton et al., 2020a; Reitl et al., 2020). In this regard, cockroaches are known to act as IH as the eggs of this cephalobaenid pentastomid develop into infective larvae (i. e. nymph) in the fat body of cockroaches (Lavoipierre and Rajamanickam, 1973; Ali and Riley, 1983; Bosch, 1986). Nevertheless, no other animal in the collection, including the male conspecific, harboured pentastomids. This suggests that the infection was most likely acquired in the wild and represents an imported case of pentastomiasis.

Interestingly, the female *R. orientalis* specimens found in this report only measured between 8 and 10 mm, compared to previous reports, stating a length of 29–61 mm (Ali et al., 1985; Walden et al., 2020). However, a recent study suggested that *R. orientalis* may show significant size variations, depending on the host species they infect. It has been speculated that this size adaptability might enable the parasite to infect a broader range of squamate DH as previously postulated (Westfall et al., 2019). Thus, this phenomenon might explain the size differences observed, being the result of the infection of a smaller lung highlighting the phenotypical plasticity of this parasite as well.

Some pentastomid genera, such as *Porocephalus* and *Armillifer* have been proven to be zoonotic, mainly infecting humans by the either consumption of raw or undercooked meat or by working very closely with reptiles especially with poor hygiene (Paré, 2008; Tappe and Büttner, 2009; Pantchev and Beck, 2013). Christoffersen and De Assis (2013) also mentioned the possibility of humans serving as accidental hosts for *R*. *orientalis*. Although, this assertion appeared somewhat vague, lacking supporting case reports from other sources, this assumption remains plausible. Nevertheless, considering that this raillietiellid species is predominantly found in snakes, and taking into account that many snake-associated pentastomids are known to have zoonotic potential, it warrants further investigations. Notably, closely-related *Raillietiella hemidactyli* has been suspected to be zoonotic in Asia, causing a human condition termed “crawling disease”, in which pentastomid larval stages acted as a *larva migrans* in the subcutaneous tissue of affected persons (Dollfus and Canet, 1954; Mendoza-Roldan et al., 2022). Furthermore, a *Raillietella* sp. larva was detected in a case of visceral pentastomiasis, co-infecting a patient with *Armillifer grandis* and *Armillifer armillatus* in the Democratic Republic of Congo (Tappe et al., 2016), which raises the intriguing question of whether other raillietiellid species might also pose zoonotic risk.

Pentastomids in reptiles typically cause subclinical infections (Flach et al., 2000) as their chitinous cuticle is coated with a self-produced surfactant thereby evading the host's immune system. As a result, hosts can survive asymptomatically for extended periods (Riley and Henderson, 1999), as in the present case. However, in cases of severe pentastomiasis, pentastomids can induce flu-like symptoms such as excessive mucus production and breathing noises (Frank, 1986). Dysecdysis in reptiles might be associated to ectoparasitic infestations (Harkewicz, 2001; Hellebuyck and Scheelings, 2018), but also being associated to severe pentastomiasis as recently reported in a king cobra (Dubey et al., 2017). Antiparasitic treatment of pentastomiasis is challenging due to the potential risks associated with the remainings of dead pentastomids in the airways of vertebrates, resulting in pro-inflammatory reactions. In particular, death of the pentastomes may lead to massive antigen release, resulting in severe complications, including suffocation, extensive organ tissue damage, and even cardiovascular distress in the reptilian host (Jacobson, 2007; Paré, 2008; Schneller et al., 2008). This might have been the reason for the final deterioration and the respiratory distress in the present case.

Previously reports have demonstrated the efficacy of ivermectin in treating pentastomiasis in geckos (Wright, 1997; Takaki et al., 2022) and monitor lizard (Flach et al., 2000). Despite being known toxic in various reptiles including some chameleons (Széll et al., 2001; Pellett et al., 2014; Panayotova-Pencheva, 2016; Knotek, 2019) ivermectine was used for treatment, because it is the sole proven medical treatment against *R. orientalis* to date (Wellehan and Walden, 2018; Sladky et al., 2023). Although there has been a case in which a group of panther chameleon (*Furcifer pardalis)* was successfully treated for strongyloidiasis using ivermectin in the same dose (0.2 mg/kg) (Heng et al., 2023) as applied in this case report. Another case of *F. pardalis* treated for microfilaremia with ivermectin also showed efficacy, however a lower dose (10 μg/kg) was administered monthly (Bielli, 2007). This highlights that species, overall health, infection severity, dosage, administration route, and other underlying diseases may affect the efficacy of the therapy. Therefore, caution is advised when using ivermectin in chameleons, starting with a lower dosage and considering potential systemic reactions triggered by parasite die-off. Additional attempts of removing pentastomids through surgery or endoscopy might have been additional treatment options, however, this has only been successfully applied in larger snake species (Foldenauer et al., 2008; Wellehan and Walden, 2018) and thus it was not attempted in the present case. Retrospectively, further research into treatment alternatives for smaller reptile species are recommended.

Despite its concerning spread, little is known about the parasite's life cycle and the potential IH that may be competent carriers. Current insights primarily come from laboratory tests (Palmisano et al., 2022).

Emerging infectious diseases (EIDs) in amphibians and reptiles, such as pathogenic fungi (e.g. *Batrachochytrium* spp., *Nannizziopsis* spp., and *Ophidiomyces ophidiicola)*, viruses (e.g. Nidovirus, Arenavirus, Ranavirus), bacteria (e.g. *Devriesea agamarum*) and parasites (e.g. *Cryptosporidium* spp.*)*, have been spreading worldwide in the last decades (Latney and Wellehan, 2013; Schilliger et al., 2023). These EIDs outbreaks affect both captive and wild animals (Williams et al., 2002; Latney and Wellehan, 2013; Schilliger et al., 2023). The rapid spread of *R*. *orientalis* as an invasive parasite in native reptile species and its infection intensity in southern Florida (Miller et al., 2020) highlights the potential threat which may pose to free-ranging wildlife in Europe and potentially worldwide. A major contributing factor is the growing animal trade and the increase in invasive species resulting from it (Keller et al., 2011; Lockwood et al., 2019). This not only impacts animal populations but also raises concerns about zoonotic potential, as EIDs carried by animals can pose risks to human health (Daszak et al., 2000, 2001).

## 4 Conclusion

The detection of a patent *R. orientalis*-induced pneumonia in a captive Meller's chameleon is most likely an incidental finding of an imported infection. Nonetheless, it most likely shows the phenotypical plasticity of this parasite by potentially infecting native insects to fulfill its life cycle. In addition, chameleons can be added as DH of *R. orientalis* besides various squamate species. The global trade of wild animals has contributed to the spread of invasive species facilitating the distribution of this parasite. As imported species can serve as natural reservoirs for EIDS, vigilant parasite monitoring and implementation of biosecurity and working safety measures are essential to reduce zoonotic risks. Increased awareness among both veterinary practicioners and public health authorities is essential in addressing neglected pentastomiasis. Significant knowledge gaps persist regarding the epizootiology of *R. orientalis* in both wild and captive reptiles, including its prevalence, pathogenic mechanisms, and host immune responses. These limitations call for further research on this emerging parasite group.

## CRediT authorship contribution statement

**Paula Sapion-Miranda:** Writing – original draft, Investigation, Visualization. **David Ebmer:** Methodology, Writing – review & editing. **Edwin Kniha:** Methodology, Investigation, Resources, Writing – review & editing. **Julia Walochnik:** Methodology, Investigation, Resources. **Saskia Dreyer:** Resources, Methodology. **Dominik Fischer:** Resources, Writing – review & editing, Methodology. **Lisa Grund:** Resources, Methodology. **Anja Taubert:** Supervision. **Carlos Hermosilla:** Writing – review & editing, Supervision, Conceptualization. **Malek J. Hallinger:** Writing – review & editing, Supervision, Conceptualization.

## Funding

This research did not receive any specific grant from funding agencies of public-, commercial-, or not-for-profit-sectors.

## Declaration of competing interest

The company Qiagen, Hilden, Germany did not play a role in the study design nor in the collection, analysis and interpretation of generated data, nor in the decision to submit the manuscript for further publication. None of the authors has any financial or personal relationship that could inappropriately influence or bias the content of the manuscript.
